# LGG/LAC-MMT combination mitigates AFB_1_-induced liver and intestinal injury in mice based on intestinal microbiota modulation

**DOI:** 10.3389/fvets.2025.1654294

**Published:** 2025-10-21

**Authors:** Jiaxin Cheng, Ying Gao, Hongming Lv, Jing Li, Xudong Sun, Tianhui An, Honglin Liu, Junqi Wang, Haifeng Zhang, Haiyan Wang, Siqi Zou, Zhao Fan, Yuanyuan Chen

**Affiliations:** ^1^College of Animal Science and Veterinary Medicine, Heilongjiang Bayi Agricultural University, Daqing High-Tech Industrial Development Zone, Daqing, China; ^2^College of Animal Science and Technology, Jilin Agricultural Science and Technology College, Jilin City, Jilin Province, China

**Keywords:** aflatoxin B1, *Lactobacillus rhamnosus*, *Lactobacillus acidophilus*, Montmorillonite, inflammatory responses, oxidative stress, intestinal microbiome

## Abstract

AFB_1_ induces hepatotoxicity and enterotoxicity. *Lactobacillus acidophilus* (LAC) and *Lactobacillus rhamnosus* (LGG), both belonging to LAB, have strong binding affinity for AFB_1_. Montmorillonite (MMT) not only adsorbs AFB_1_ but also serves as a carrier for LAB, thereby enhancing their colonization ability and prolonging their survival. Despite the unclear effects of LGG/LAC-MMT combination on AFB_1_-induced tissue injury and intestinal microbiota disruption, this study aimed to determine whether it could effectively alleviate tissue damage from AFB_1_ exposure and enhance LAB colonization capacity in mouse intestines. Separately, LGG (2 × 10^9^ cfu/mL) and LAC (2 × 10^9^ cfu/mL) were combined with MMT (0.5 mg/kg), and the AFB_1_-intoxicated mice were gavaged with the mixtures for 4 weeks. Findings suggested that LGG, LAC, and MMT supplementation restored oxidative stress and inflammatory caused by AFB_1_ to some degree. Furthermore, they altered the intestinal microbiota structure, enhancing the colonization ability of LABs, thereby alleviating liver and intestinal injury. The combination of LGG/LAC-MMT was more effective, especially LAC-MMT. Overall, LGG/LAC-MMT exhibits a synergistic effect and can effectively ameliorate AFB_1_-induced tissue injury and intestinal microbiota disorder.

## 1 Introduction

As a well-recognized Class I carcinogen, aflatoxin B1 (AFB_1_) represents the most prevalent and toxic aflatoxin subtype (1, 2). Both ruminants and monogastric animals are vulnerable to AFB_1_-induced damage, which can lead to severe health issues and even lethal outcomes (3–5). Animals consuming AFB_1_ may exhibit diverse toxicities leading to organ and tissue injury, including but not limited to hepatotoxicity, enterotoxicity, nephrotoxicity, splenotoxicity, pulmonary toxicity, and neurotoxicity (6–9). Consequently, it is imperative to conduct extensive studies on mitigating AFB_1_ toxicity.

Hepatotoxicity and enterotoxicity frequently occur upon AFB_1_ exposure. AFB_1_ affects the liver as its primary target organ (10). Upon animal ingestion, AFB_1_-contaminated feed is absorbed through the gastric and intestinal mucosa, ultimately leading to liver injury (11). Studies have shown that oral administration of AFB_1_ to rats for just 2 consecutive weeks can cause significant damage to liver tissue (6, 12). The potential mechanisms by which AFB_1_ induces hepatotoxicity lie in oxidative stress and inflammatory responses (13). In rodent studies of AFB_1_ exposure, AFB_1_ was shown to induce a hepatic oxidative stress-inflammatory cascade, resulting in elevated oxidative stress biomarkers and pro-inflammatory mediators, while depleting enzymatic antioxidant reserves (14–16). This cascade ultimately results in liver injury, leading to diminished liver function indicators (15). Intestinal injury is also accompanied by oxidative stress and inflammatory responses (2), with concomitant effects on the intestinal microbiota. Research evidence suggests that AFB_1_ compromises intestinal barrier integrity, resulting in a decline of commensal microbiota and proliferation of pathogenic bacteria (17, 18). A dysregulated intestinal microbiome can impair host health and provide a foundation for disease development.

In recent years, investigations into the detoxifying efficacy of biocontrol agents against AFB_1_ have proliferated. Lactic acid bacteria (LAB), such as *Lactobacillus acidophilus* (LAC) and *Lactobacillus rhamnosus* GG (LGG), have an *in vitro* binding ability to AFB_1_ ranging from 64.56% to 96.58% (19). Moreover, LAC and LGG have been shown to mitigate AFB_1_-induced hepatic and intestinal damage across multiple animal models (such as fish, rats and chicken), while also reversing AFB_1_-mediated growth suppression (20–22). However, environmental factors significantly influence the AFB_1_ detoxifying capacity of LAB. The principal influencing factor is that the adhesion capacity of LAB to the intestine and their binding ability for AFB_1_ are surface-associated, resulting in a diminished colonization capability of LAB (23). Additionally, research has validated that intestinal mucus influences the adsorption of AFB_1_ by LAB *in vivo* (24). Furthermore, the gastrointestinal environment is relatively harsh, and the introduction of exogenous LAB *in vivo* may reduce their survival rates, thereby impacting the detoxifying efficacy of LAB against AFB_1_ (25). Consequently, using a carrier to deliver LAB *in vivo* has been proposed to address this issue. Montmorillonite (MMT) is a commonly employed silicate that can serve as a carrier for LAB. Studies have demonstrated that the LAB-MMT combination can effectively maintain LAB viability within the intestinal tract of mice (26). Simultaneously, MMT, either in its native form or modified, can serve as an effective adsorbent for AFB_1_ both *in vivo* and *in vitro*, and inhibit intestinal pathogenic bacteria (27–30). Therefore, the *in vivo* combination of LAB-MMT has significant potential for minimizing AFB_1_ toxicity in animals. However, research on the LAB-MMT combination remains limited.

In summary, this study will administer LAC/LGG-MMT to AFB_1_-exposed mice. It will *in vivo* investigate the detoxification effects of different LAB-MMT combinations in AFB_1_-exposed mice *in vivo*.

## 2 Materials and methods

This study gained ethical approval from Heilongjiang Bayi Agricultural University's Science and Technology Ethics Committee (Approval number: DWKJXY2024034).

### 2.1 Preparation of AFB_1_ and strains culture

AFB_1_ was purchased from FERMENTEK Ltd. (Qingdao, China). An AFB_1_ stock solution (1 mg/mL) was prepared using 10% dimethyl sulfoxide (DMSO, Fisher) as the solvent. It was diluted with sterile water before gavage and administered orally at a dose of 400 μg/kg based on mouse body weight. The LAC and LGG strains were purchased from BeNa Culture Collection Biotechnology Co., Ltd. (Hebei, China). The strains were activated according to the instructions, subsequently streaked three times onto MRS agar plates, and incubated at 37 °C for 16 h. Purified LAC and LGG bacterial fluids were centrifuged in a high-speed cryo-centrifuge (4 °C, 2,000 rpm, 10 min). The bacteria were washed twice with PBS (pH = 7.4) and resuspended in the same buffer. A UV spectrophotometer was employed to adjust the bacterial suspension to the target concentration. The final viable bacteria concentration was adjusted to 2 × 10^9^ cfu/mL. MMT is supplied by American Anmuran International Co., Ltd (Shenzhen, China). Its main components are 70% calcium-based MMT (calcium content of 0.5% to 1.5%), 15% amorphous hydrated silica, and 15% other minerals. An MMT solution was prepared by dissolving 0.5 mg/kg MMT in 1 mL of sterile distilled water. Additionally, the LAC/LGG-MMT complex was prepared by mixing 0.5 mg/mL MMT directly with 2 × 10^9^ cfu/mL LGG and LAC suspensions.

### 2.2 Animals, experimental design and sample acquisition

Four-week-old male Balb/c mice [18–22 g; Harbin Medical University Laboratory Animal Division, SCXK (BK) 2024-002] were maintained under standardized conditions: 20 ± 2 °C, 50% ± 5% relative humidity, 16 h light/dark cycle, with unrestricted access to food and water.

After a one-week adaptation period, 80 mice (*n* = 80) were randomly divided into eight groups with 10 mice per group: Group C received 400 μL sterile distilled water; Group D received 400 μL DMSO; Group A were orally administered 400 μg/kg AFB_1_; Group A+M received 400 μL AFB_1_ and 0.5 mg/kg MMT solution; Group A+L1 received 400 μg/kg AFB_1_ and 2 × 10^9^ cfu/mL LGG; Group A+L2 received 400 μg/kg AFB_1_ and 2 × 10^9^ cfu/mL LAC; Group A+M+L1 received 400 μg/kg AFB_1_, 0.5 mg/kg MMT solution, and 2 × 10^9^ cfu/mL LGG; Group A+M+L2 received 400 μg/kg AFB_1_, 0.5 mg/kg MMT solution, and 2 × 10^9^ cfu/mL LAC. After the four-week experimental period, mice were sacrificed by eyeball blood sampling and neck removal, and then liver, colon, and cecal contents were collected.

### 2.3 Histopathological observation

Tissues of the liver and jejunum of mice were fixed in 4% paraformaldehyde for 24 h, processed into paraffin-embedded blocks, cut into 5-μm sections, stained with H&E, and examined under a light microscope to capture and store histopathological images for analysis.

### 2.4 Measurement of serum inflammatory factors and blood biochemical indices

Serum was separated to measure inflammatory cytokines (IL-1β, TNF-α, IL-6, IFN-γ, IL-2, and IL-8) using ELISA kit (Nanjing Jiancheng Biotechnology Co., Ltd., Nanjing, China) according to established protocols, with liver function indices including alanine aminotransferase (ALT), aspartate aminotransferase (AST), alkaline phosphatase (ALP), total protein (TP) and albumin (ALB) assayed via a fully automated biochemical analyzer (Shenzhen Rayto Life and Analytical Sciences Co., Ltd., Shenzhen, China).

### 2.5 Determination of antioxidant enzyme content in liver and intestine

Liver and intestinal tissues were minced and loaded into centrifuge tubes. Pre-cooled PBS was added to tubes, samples were homogenized and centrifuged at 4 °C (1,700 rpm, 10 min), supernatant was discarded, malondialdehyde (MDA) content was assayed using Shanghai Langton Bioscience ELISA kit per protocol, and remaining supernatant was diluted with PBS to measure total protein via the company's BCA kit. The levels of superoxide dismutase (SOD), catalase (CAT), glutathione (GSH), and glutathione reductase (GR) were assayed using ELISA kits from Shanghai Langton Bioscience Co., Ltd. (Shanghai, China) as per the provided protocols.

### 2.6 Real-time RT-PCR analysis for mRNA levels of liver inflammatory factors

Using TRIzol reagent (Invitrogen Corporation, CA, USA), total RNA was extracted from murine liver tissues as per the manufacturer's standard procedure. Then, the high-speed centrifuge was then pre-cooled to 4 °C for centrifugation (12,000 rpm, 10 min). Sample RNA concentrations and OD_260/280_ values were determined using a UV spectrophotometer. Following cDNA synthesis, single-stranded products were subjected to qRT-PCR for quantification of target gene mRNA levels. Table 1 provides the detailed primer sequences. By applying the 2^−Δ*ΔCt*^ method, relative target gene expression was calculated and normalized to β-actin (housekeeping gene).

**Table 1:** Primer sequences used in this study.

**Gene**	**Primer sequence (5' → 3^′^)**	**Product size (bp)**
β-actin	F: 5′-GAGACCTTCAACACCCCAGC-3′	263 bp
	R: 5′-ATGTCACGCACGATTTCCC-3′	
IL-1β	F: 5′-AGCTTCAAATCTCGCAGCAG-3′	72 bp
	R: 5′-TCTCCACAGCCACAATGAGT-3′	
TNF-α	F: 5′-CTCATGCACCACCATCAAGG-3′	96 bp
	R: 5′-ACCTGACCACTCTCCCTTTG-3′	
IL-6	F: 5′-CCAAGAGGTGAGTGCTTCCC-3′	127 bp
	R: 5′-CTGTTGTTCAGACTCTCTCCCT-3′	
IL-10	F: 5′-GCTCTTACTGACTGGCATGAG-3′	109 bp
	R: 5′-CGCAGCTCTAGGAGCATGTG-3′	
IL-2	F: 5′-CCAAGCAGGCCACAGAATTG-3′	199 bp
	R: 5′-GCTGACTCATCATCGAATTGGC-3′	
IL-8	F: 5′-GAGACCTTCAACACCCCAGC-3′	106 bp
	R: 5′-ATGTCACGCACGATTTCCC-3′	

### 2.7 16S rRNA sequencing

The TIANGEN Fecal Genomic DNA Extraction Kit (TIANGEN Biochemical Technology Co., Ltd., Beijing, China) was used to isolate fecal microbial DNA following the 16S rRNA-specific protocol. The integrity was verified by agarose gel electrophoresis, while the quality was determined by NanoDrop 2000. Using bacterial primers 338F 5′-ACTCCTACGGGAGGCAGCAG3′) and 806R 5′-GGACTACHVGGGTWTCTAAT3′), we amplified the 16S rRNA V3-V4 region via PCR. After visualization of the amplification products by 2% agarose gel electrophoresis, the products were extracted using a DNA gel recovery kit (Shanghai Bioengineering Co., Ltd., Shanghai, China). Beijing Nuohe Co., Ltd. was entrusted with the sequencing of the PCR products.

### 2.8 High performance liquid chromatography (HPLC) for AFB_1_ in the feces

Feces were collected aseptically from the colon and cecum of each group of mice. The feces from 10 mice of the same group were mixed, so that the total wet weight of feces in each group amounted to 4-5 g to form a pooled material (31), which can reduce individual differences and to meet the DNA extraction requirements. Then the pooled material of feces was mixed with 10 mL 0.1% acidified acetonitrile, vortexed and sonicated (10 min each, 4 °C), then centrifuged at 13,000 rpm for 10 min at 4 °C. Supernatant (2 mL) was loaded onto a PRiMEHLB column, eluted with acetonitrile (2 mL), dried under nitrogen, reconstituted in acetonitrile (1 mL), filtered (0.22 μm), and analyzed by UPLC-MS/MS on a TSK GEL-ODS100V column (150 × 2.1 mm, 5 μm).Gradient elution: 0.3 mL/min flow rate, 40 °C column temperature, mobile phases A (0.1% formic acid in water) and B (acetonitrile), 10-μL injection. LOD (S/N ≥3) and LOQ (S/N ≥10) were set as per standard protocols.

### 2.9 Statistical analysis

Microsoft Excel was used for preliminary statistical analysis of the experimental data, and SPSS 17.0 software was used for data processing and One-way ANOVA was used to analyze the significance of each group, and the post-event comparison method is the Duncan. *P* < 0.05 was considered statistically significant. The results were expressed as mean ± SD. GraphPad Prism 7.0 software and OriginPro 2024b were used to draw line, bar charts and box plots. The ACE, shannon, simpson, chao1 and coverage indices were calculated using QIIME2 software. Cumulative species curve, Ternary phase diagram and dilution curves were plotted using R package.

## 3 Results

### 3.1 Growth performance

**Figure 1:**
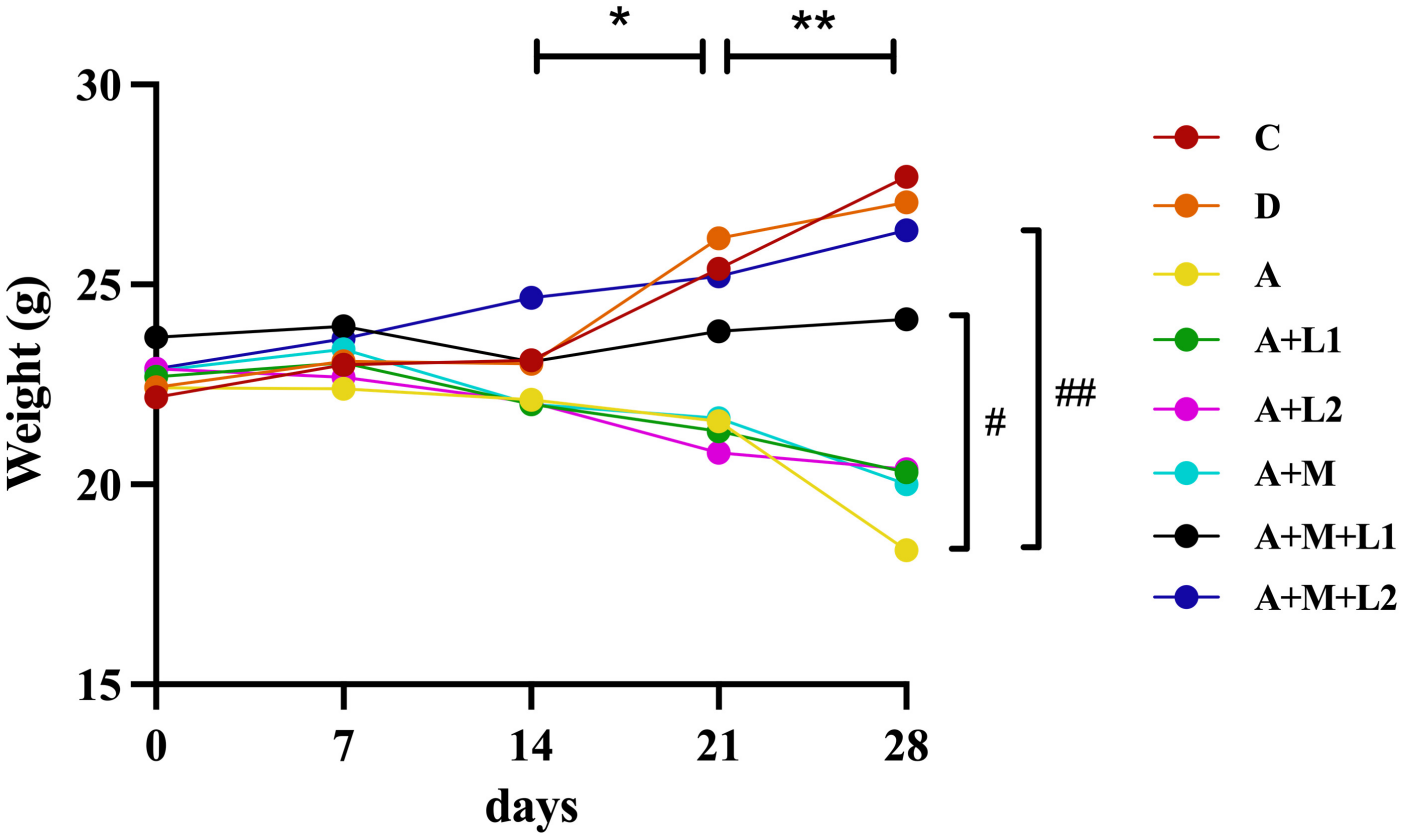
Weekly mice body weight measurements. *P < 0.05, **P < 0.01: AFB_1_/monotherapy vs. control group. ^**#**^*P* < 0.05, ^**##**^*P* < 0.01: AFB_1_+LGG-MMT vs. AFB_1_ group.

Weekly fluctuations in body weight among mice were analyzed (Figure 1). Groups showed no significant differences in initial body weights. Body weights of Group C and Group D were similar over 4 weeks. Conversely, Group A exhibited persistent progressive body weight reduction, significantly lower at weeks 3 (*P* < 0.05) and 4 (*P* < 0.01) relative to Group C. Groups A+L1, A+L2, and A+M showed progressive decreases, with *P* < 0.05 at week 3 and *P* < 0.01 at week 4 vs. Group C. After combining LGG or LAC with MMT, respectively, body weights in Groups A+M+L1 (*P* < 0.05) and A+M+L2 (*P* < 0.01) were significantly increased, exhibiting substantial growth in the third and fourth weeks compared with Group A. Throughout the study, Group A+M+L2 demonstrated a steady increase in body weight, which trended toward the values of Group C.

### 3.2 Liver histopathological observations

**Figure 2:**
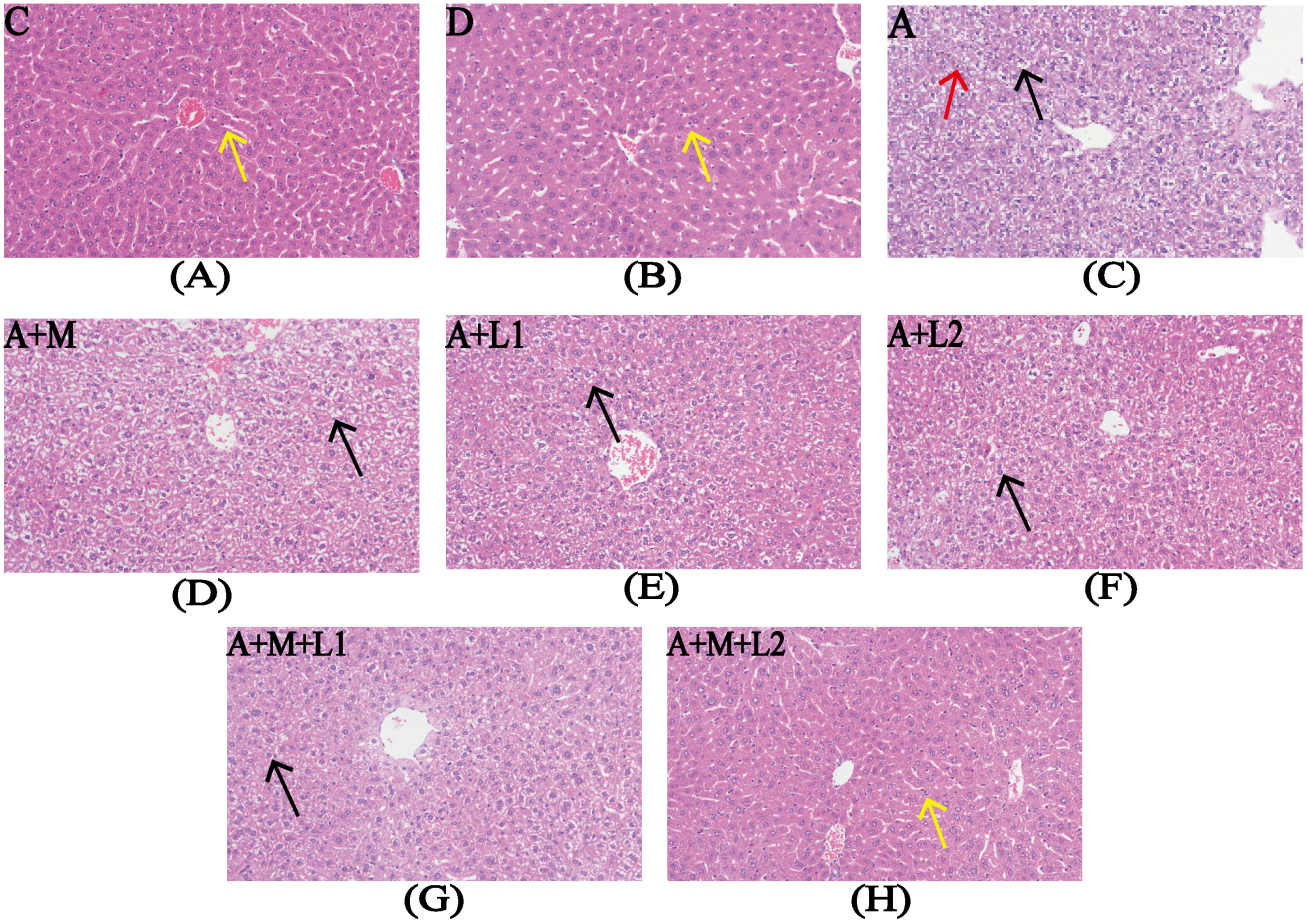
Liver histopathology after treatments. Representative images from each group (200×magnification). Yellow arrows: hepatic sinusoidal macrophages; red arrows: nuclear condensation/lysis; black arrows: hepatocyte vacuolar degeneration. Figure legend descriptions: **(A)** Group C, **(B)** Group D, **(C)** Group A, **(D)** Group A+M, **(E)** Group A+L1, **(F)** Group A+M, **(G)** Group A+M+L1, **(H)** Group A+M+L2.

Liver histopathology was assessed to determine AFB_1_-induced damage in murine hepatic tissue and the therapeutic efficacy of LGG/LAC-MMT (Figure 2). The liver histological structure of the mice in Groups C and D was predominantly normal, with intact hepatocyte architecture and clearly discernible hepatic sinusoidal macrophages (Figures 2A, B). Conversely, AFB_1_ exposure resulted in significant hepatic damage, characterized by disrupted hepatocyte architecture, pronounced inflammatory cell infiltration, extensive edema, vacuolar degeneration of hepatocytes, and nuclear condensation and lysis (Figure 2C). Unlike Group A, Groups A+L1, A+L2, and A+M failed to significantly reduce AFB_1_-induced liver lesions (Figures 2D–F). In contrast, Groups A+M+L2 and A+M+L1 partially mitigated hepatic damage, such as edema and necrosis, while preserving hepatocyte architecture and showing no inflammatory cell infiltration (Figures 2G, H).

### 3.3 Intestinal tract histopathological observations

**Figure 3:**
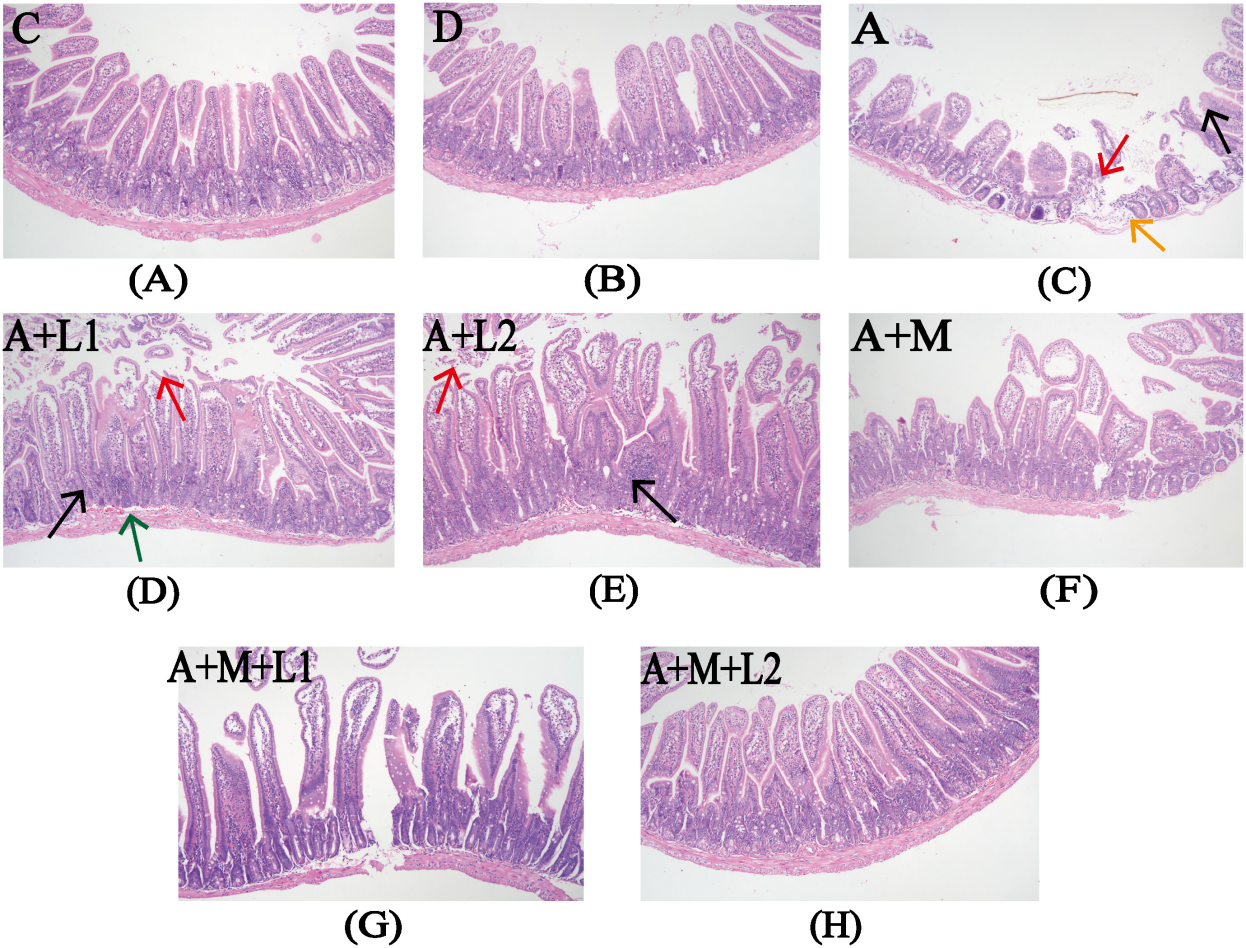
Intestinal histopathology after treatments. Representative images from each group (200×). Red arrows: exfoliated intestinal epithelial cells; orange arrows: submucosal edema; black arrows: inflammatory cell infiltration; green arrows: submucosal bleeding. Figure legend descriptions: **(A)** Group C, **(B)** Group D, **(C)** Group A, **(D)** Group A+M, **(E)** Group A+L1, **(F)** Group A+M, **(G)** Group A+M+L1, **(H)** Group A+M+L2.

Figure 3 illustrates the alterations in the jejunal tissue of mice subjected to various combinations of AFB_1_, MMT, LGG, and LAC. In Groups C and D, intestinal villi had regular histological structures, with no loose or edematous, necrotic epithelial cell degeneration and no inflammatory cell infiltration (Figures 3A, B). The intestinal barrier of mice in group A was impaired, as exhibited by shortened intestinal villi, mucosal epithelial cell detachment, submucosal edema, and minimal inflammatory cell infiltration (Figure 3C). Compared with Group A, Groups A+L1 and A+L2 showed no significant improvement (Figures 3D, E). In contrast, the impairment of intestinal barrier function was ameliorated in the Groups A+M, A+M+L1, and A+M+L2. The intestinal tissue structure normalized, exhibiting regularly distributed villi and an absence of pathological alterations (Figures 3F–H).

### 3.4 Serum liver function indicators detection

Table 2 illustrates the liver function indicators in the serum of each group. AFB_1_ induced significant elevation of ALT and AST levels and significant reduction of TP and ALB levels (*P* < 0.001). The levels of liver function indicators in Groups A+M, A+L1, A+L2 and A+M+L1 were improved to different degrees. Group A+M+L2 had the best effect, where the levels of AST, ALP, TP and ALB did not differ from Group C (*P* < 0.001).

**Table 2:** Effect of AFB_1_ on serum liver function indices and LAB-MMT-mediated improvement.

**Liver function (U ·L^−1^)**	**Groups**							
	**C**	**D**	**A**	**A**+**M**	**A**+**L1**	**A**+**L2**	**A**+**M**+**L1**	**A**+**M**+**L2**
ALT	25.54 ± 0.76^d^	26.49 ± 1.41^d^	66.36 ± 1.24^a^	64.88 ± 1.08^a^	65.91 ± 1.05^a^	58.65 ± 1.28^b^	50.18 ± 0.84^c^	47.90 ± 0.99^c^
AST	132.16 ± 2.06^d^	131.25 ± 1.31^d^	205.56 ± 1.93^a^	170.62 ± 2.07^b^	197.92 ± 2.72^a^	179.23 ± 1.88^b^	156.19 ± 2.04^c^	137.14 ± 3.05^d^
ALP	156.96 ± 2.23^d^	158.97 ± 1.36^d^	178.38 ± 2.86^a^	169.88 ± 2.09^b^	169.67 ± 1.67^b^	162.10 ± 1.55^c^	158.77 ± 1.50^d^	156.04 ± 1.72^d^
TP	56.67 ± 2.58^d^	57.38 ± 3.10^d^	33.62 ± 1.98^a^	41.09 ± 1.45^b^	40.66 ± 1.15^b^	45.08 ± 0.62^b^	51.56 ± 1.33^c^	58.80 ± 0.90^d^
ALB	31.86 ± 0.86^d^	33.10 ± 1.59^d^	19.98 ± 1.63^a^	23.75 ± 1.16^b^	24.91 ± 1.16^b^	23.29 ± 1.05^b^	29.84 ± 1.19^c^	34.21 ± 0.77^d^

^a^Values are Mean ± SD (n = 10). ALT, aminotransferase; AST, aspartate aminotransferase; ALP, alkaline phosphatase; TP, total protein; ALB, albumin.

^a, b, c, d^Different letters = significant differences (*P* < 0.05).

### 3.5 Serum inflammatory factor detection

**Figure 4:**
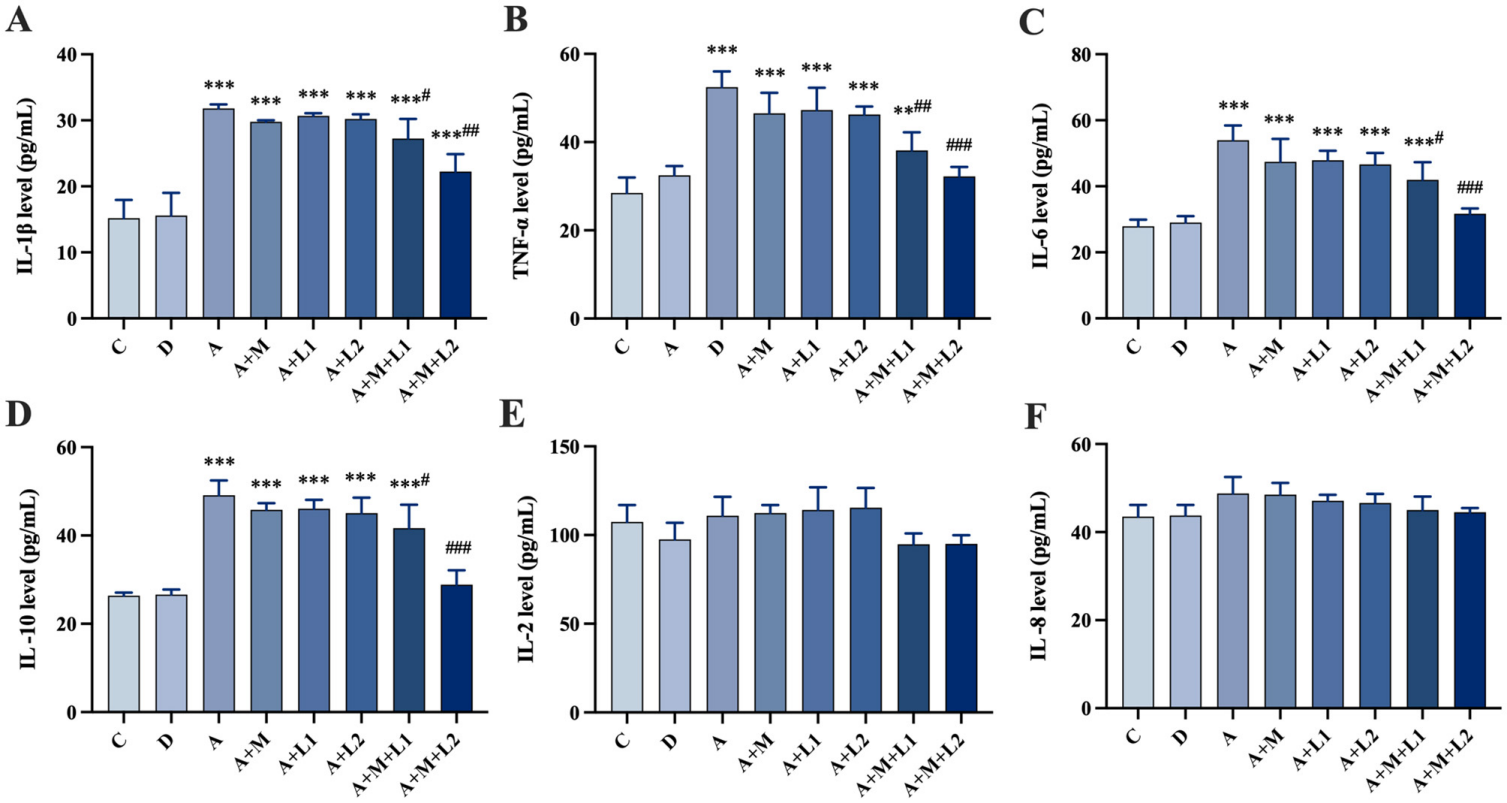
Mice serum cytokines: **(A)** IL-1β, **(B)** TNF-α, **(C)** IL-6, **(D)** IL-10, **(E)** IL-2, **(F)** IL-8. Values are mean ± SD. *, **, ***P < 0.05, 0.01, 0.001 vs. control group. ^#^, ^##^, ^###^*P* < 0.05, 0.01, 0.001 vs. AFB_1_ group.

Figure 4 shows serum inflammatory factor profiles. Compared with Group C, IL-1β, TNF-α, IL-6 and IL-10 were significantly upregulated in Groups A, A+M, A+L1, and A+L2 (*P* < 0.001, Figures 4A–D). Group A+M+L1 had lower IL-1β (*P* < 0.05) and TNF-α (*P* < 0.01) than Group A (Figures 4A, B). Group A+M+L2 demonstrated significant reductions in IL-1β (*P* < 0.01), TNF-α, IL-6, and IL-10 (*P* < 0.001, Figures 4A–D). IL-2 and IL-8 levels were not significantly different across groups.

### 3.6 Liver antioxidant enzyme detection

**Figure 5:**
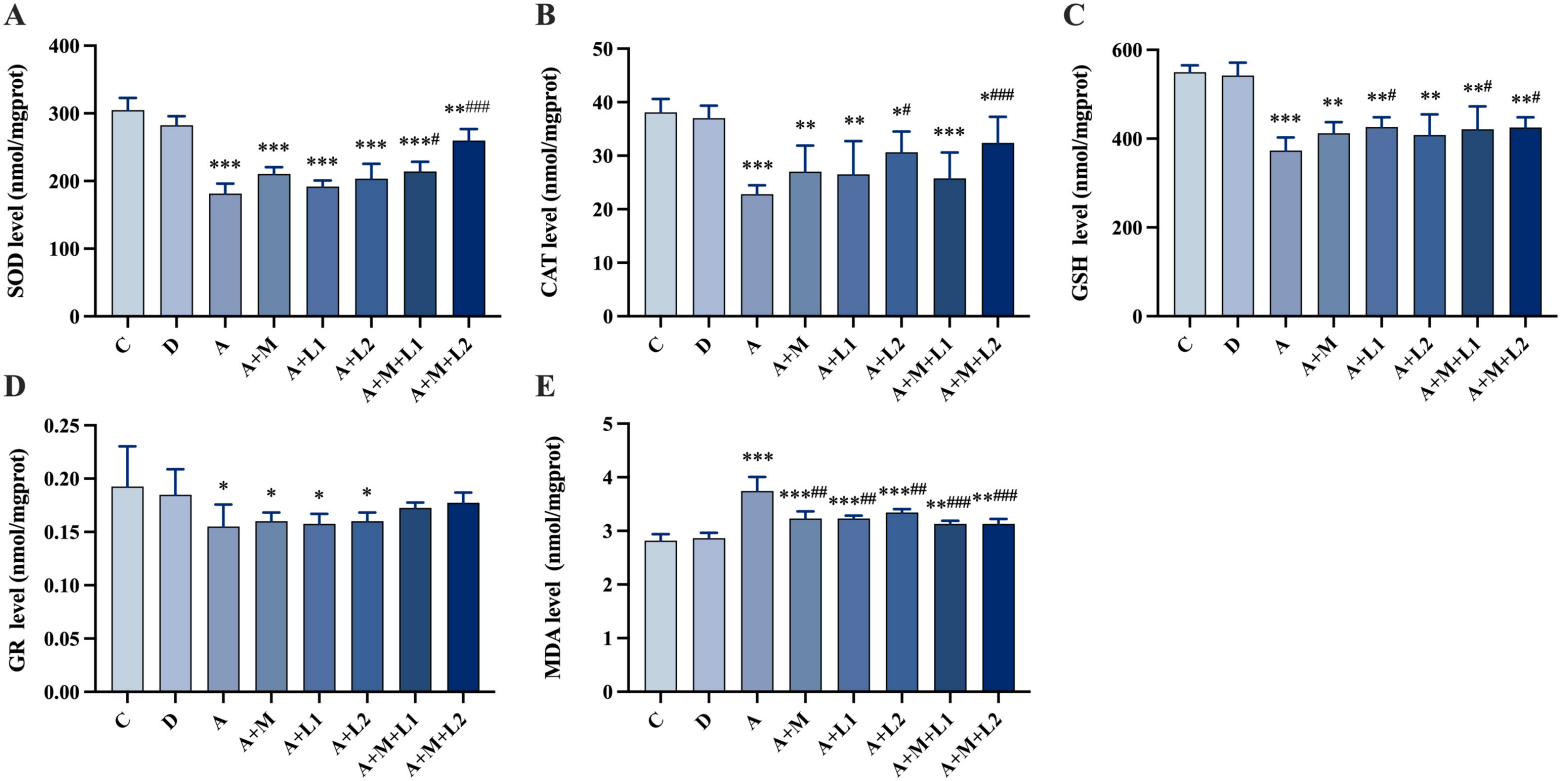
Antioxidant enzymes and MDA in mice liver. **(A)** SOD, **(B)** CAT, **(C)** GSH, **(D)** GR and **(E)** MDA. Values are mean ± SD. *, **, ***P < 0.05, 0.01, 0.001 vs. control group. ^#^, ^##^, ^###^*P* < 0.05, 0.01, 0.001 vs. AFB_1_ group.

Figure 5 shows the changes in antioxidant enzyme levels within liver tissues across groups. Group A had lower SOD, CAT, GSH (*P* < 0.001; Figures 5A–C), reduced GR (*P* < 0.05; Figure 5D), higher MDA (*P* < 0.001; Figure 5E). Compared to Group A, Groups A+L1 and A+M had comparable antioxidant enzyme levels (*P* > 0.05, Figures 5A–D), yet exhibited lower MDA levels (*P* < 0.01, Figure 5E). Group A+L2 displayed increased CAT content and reduced MDA content (*P* < 0.05, Figures 5B, E). Meanwhile, Group A+M+L1 enhanced SOD and GSH contents (*P* < 0.05, Figures 5A, C), and Group A+M+L2 significantly elevated SOD, CAT, and GSH (*P* < 0.001, *P* < 0.01, *P* < 0.05, Figures 5A–C). GR content remained unchanged in treatment groups (*P* > 0.05, Figure 5D), while both A+M+L1 and A+M+L2 decreased MDA levels relative to Group A (*P* < 0.001, Figure 5E). Notably, these combined treatments, though superior to single components, still showed higher MDA content than Group C (*P* < 0.01, Figure 5E).

### 3.7 Liver inflammatory factor mRNA detection

**Figure 6:**
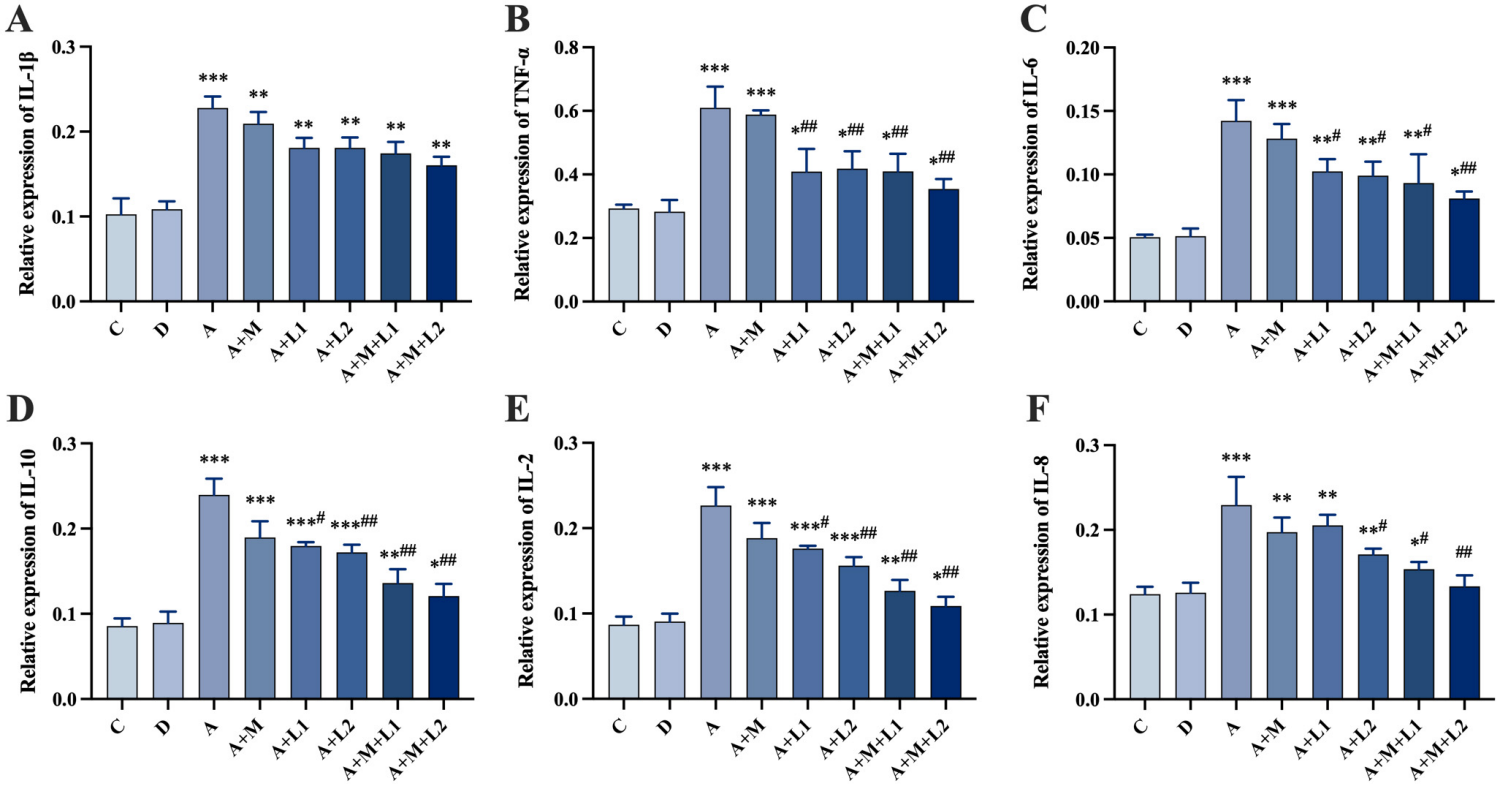
Liver inflammatory cytokine mRNA levels. **(A)** IL-1β, **(B)** TNF-α, **(C)** IL-6, **(D)** IL-10, **(E)** IL-2, **(F)** IL-8. Values are mean ± SD. *, **, ***P < 0.05, 0.01, 0.001 vs. control group. ^#^, ^##^, ^###^*P* < 0.05, 0.01, 0.001 vs. AFB_1_ group.

Figure 6 presents the hepatic inflammatory factor mRNA expression profiles in mice. Group A showed a significant up-regulation of six inflammatory factor mRNAs compared to Group C (*P* < 0.001, Figures 6A–F). Compared with Group A, Group A+L1 showed significantly lower mRNA levels of TNF-α (*P* < 0.01, Figure 6B) and IL-6, IL-10, and IL-2 (*P* < 0.05, Figures 6C–E). TNF-α, IL-6, IL-8, IL-10, and IL-2 mRNA levels were significantly down-regulated in Group A+L2 (*P* < 0.01, Figures 6B, D, E; *P* < 0.05, Figures 6C, F). Additionally, these factors were significantly decreased in Group A+M+L1 (*P* < 0.05, Figures 6C, F; *P* < 0.01, Figures 6B, D, E), with more pronounced effects in Group A+M+L2 (*P* < 0.01, Figures 6B–F).

### 3.8 Intestinal antioxidant enzyme

**Figure 7:**
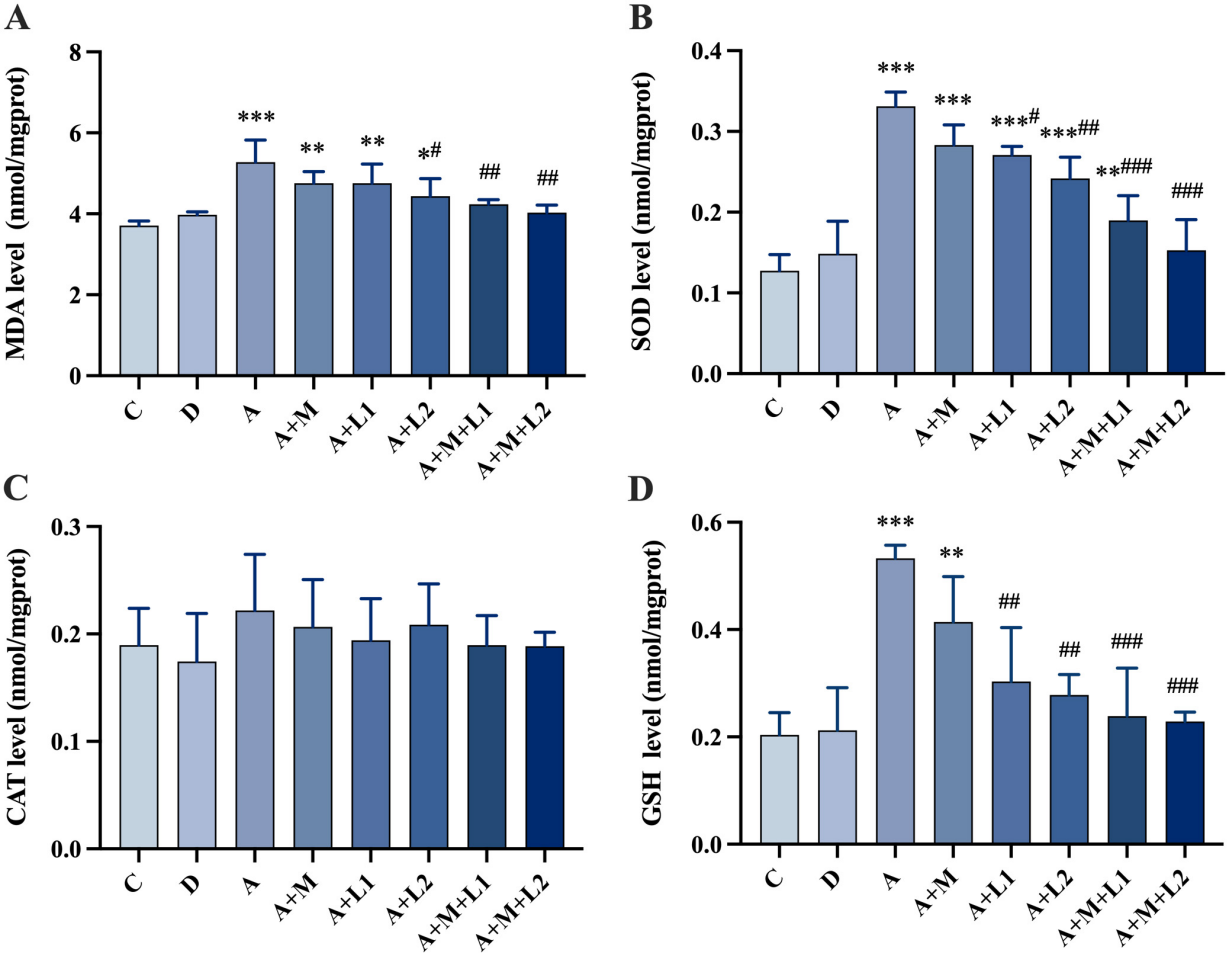
Antioxidant enzyme changes in mice intestinal injury. **(A)** MDA; **(B)** SOD; **(C)** CAT; **(D)** GSH. Values are mean ± SD. *, **, ***P < 0.05, 0.01, 0.001 vs. control group. ^#^, ^##^, ^###^*P* < 0.05, 0.01, 0.001 vs. AFB_1_ group.

Figure 7 illustrates the results of alterations in oxidative stress indices within the intestinal tissues. CAT activity showed no significant changes (*P* > 0.05, Figure 7C). MDA and SOD/GSH contents in Group A were significantly increased (*P* < 0.001, Figures 7A, B, D) compared with Group C. Group A+L1 had statistically significant decreases in SOD (*P* < 0.05, Figure 7B) and GSH (*P* < 0.01, Figure 7D) contents compared with Group A. Group A+L2 demonstrated a significant decrease in MDA (*P* < 0.05, Figure 7A) and a highly significant reduction in both SOD and GSH contents (*P* < 0.01, Figures 7B, D). Groups A+M+L1 and A+M+L2 exhibited a highly significant reduction in MDA (*P* < 0.01, Figure 7A) and SOD/GSH contents (*P* < 0.001, Figures 7B, D).

### 3.9 Alterations in the microbiota of cecal contents in mice

**Figure 8:**
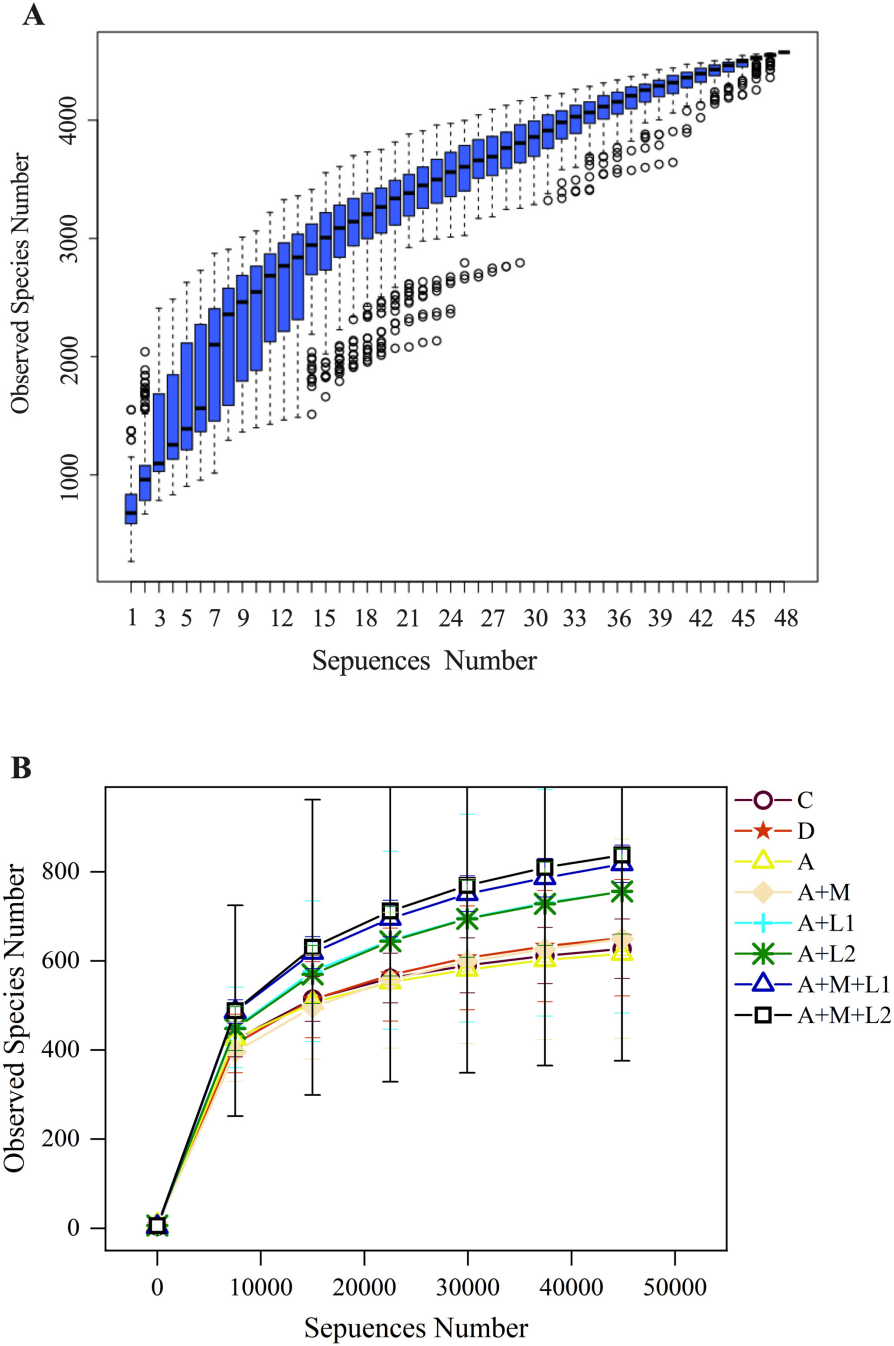
Cumulative species curve **(A)** in cecal content samples of mice and species alpha diversity dilution curve **(B)**.

The experiment began with an analysis of the cumulative and alpha diversity in cecal content samples from mice (Figure 8). The species accumulation curve (Figure 8A) illustrates that the curve increases with larger sample sizes and ultimately levels off. This signifies that the sample size was adequate for subsequent assessments of species richness and diversity. Dilution curves were generated by correlating the volume of sequencing data obtained with the respective number of species (Figure 8B). The curve exhibits a tendency to flatten with increased sequencing depth, indicating that the sequencing data accumulation was stable and sufficient. Furthermore, even with larger data volumes, only a limited number of new OTUs were observed, which would not affect the experiment.

#### 3.9.1 Alterations in intergroup disparities in the alpha diversity index of cecal microbiota

Alpha diversity indices were employed to quantify within-sample community richness and diversity. Sequencing coverage exceeded 99.70% for all groups (Table 3), showing that the depth captured all sample species. Notable discrepancies in ACE/Chao1 metrics were observed between Group A and C, coinciding with shifts in murine intestinal microbial richness and diversity. When LAB or MMT was applied independently, ACE and Simpson indices showed no significant differences from Group A (*P* > 0.05), while Shannon and Chao1 indices were substantially elevated (*P* < 0.05). Significantly, following the incorporation of the LGG/LAC-MMT combination, all three indices (ACE, Simpson, and Chao1) exhibited substantial increases (*P* < 0.05), with LAC-MMT demonstrating stronger effects on intestinal microbiota richness and diversity in mice.

**Table 3:** Analysis of α diversity of cecal flora in each group.

**Groups**	**ACE indices**	**Shannon indices**	**Simpson indices**	**Chao1 indices**	**Coverage rate**
C	855.42	6.769	0.972	730.48	0.997
D	835.061	6.435	0.961	708.606	0.997
A	661.894^*^	5.825	0.907	656.31^*^	0.997
A+M	735.463	5.954	0.954	833.7^#^	0.997
A+L1	718.994	5.9	0.958	730.48^#^	0.997
A+L2	672.607	6.117^#^	0.952	817.997^#^	0.998
A+M+L1	929.05^#^	6.251^#^	0.961	905.981^*#^	0.998
A+M+L2	939.741^##^	6.407^##^	0.972	916.49^*#^	0.998

^*^P < 0.05: Control-vs-other groups. ^#^(*P* < 0.05), ^##^(*P* < 0.01): AFB_1_-vs-AFB_1_+Lactobacillus.

#### 3.9.2 Variations in the microbiota composition within the cecal contents

**Figure 9:**
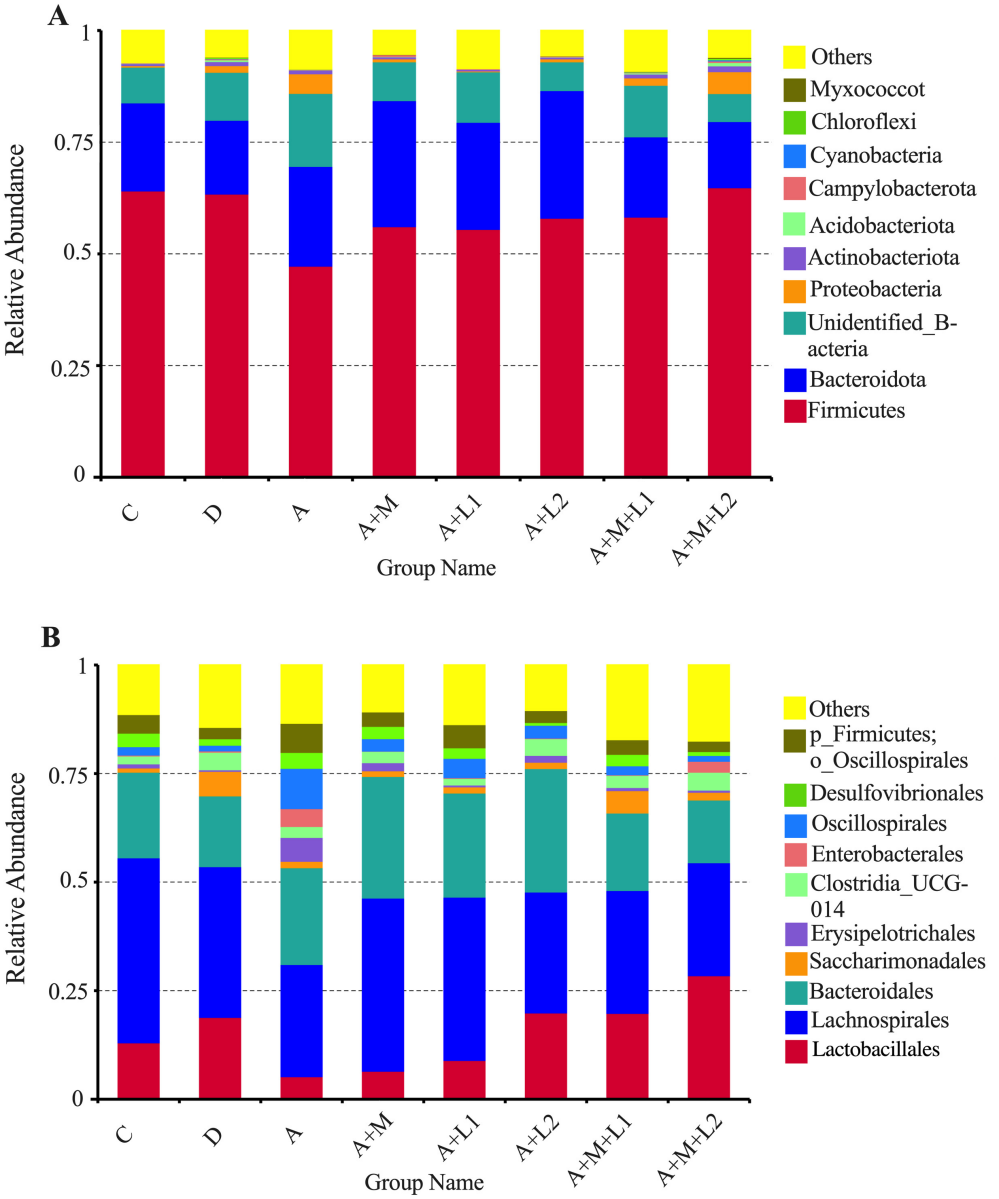
Bacterial phylum **(A)** and order **(B)** abundances in mice cecum.

The effects of LAB and MMT on cecal microbiota abundance at phylum and order levels were analyzed in mice (Figure 9). At the Phylum level (Figure 9A), more than 80% of the intestinal microbiota in mice comprises predominant bacterial phyla, including *Firmicutes, Bacteroidota, Proteobacteria, Actinobacteria*, and *Cyanobacteria*. A notable reduction in Firmicutes relative abundance was observed from Group C (64.13%) to Group A (47.29%). In contrast, the relative abundance of *Bacteroidota* increased from 19.71% (Group C) to 22.36% (Group A). Moderate increases in beneficial microbial communities were observed in A+M, A+L1, A+L2, and A+M+L1 vs. Group A, with a marked rise in Firmicutes abundance specific to A+M+L2.

In Group A+M+L2, *Lactobacillales* had the greatest relative abundance (23.27%) at the Order level (Figure 9B), followed by Groups A+L2 (14.69%) and A+M+L1 (14.58%), while Group A had the lowest (7.82%). Moreover, the predominant bacterial groups, including *Lachnospirales* and *Bacteroidales*, showed a tendency to be consistent with the changes in the relative abundance of *Lactobacillales*.

#### 3.9.3 Intestinal antioxidant capacity and function enzymes

**Figure 10:**
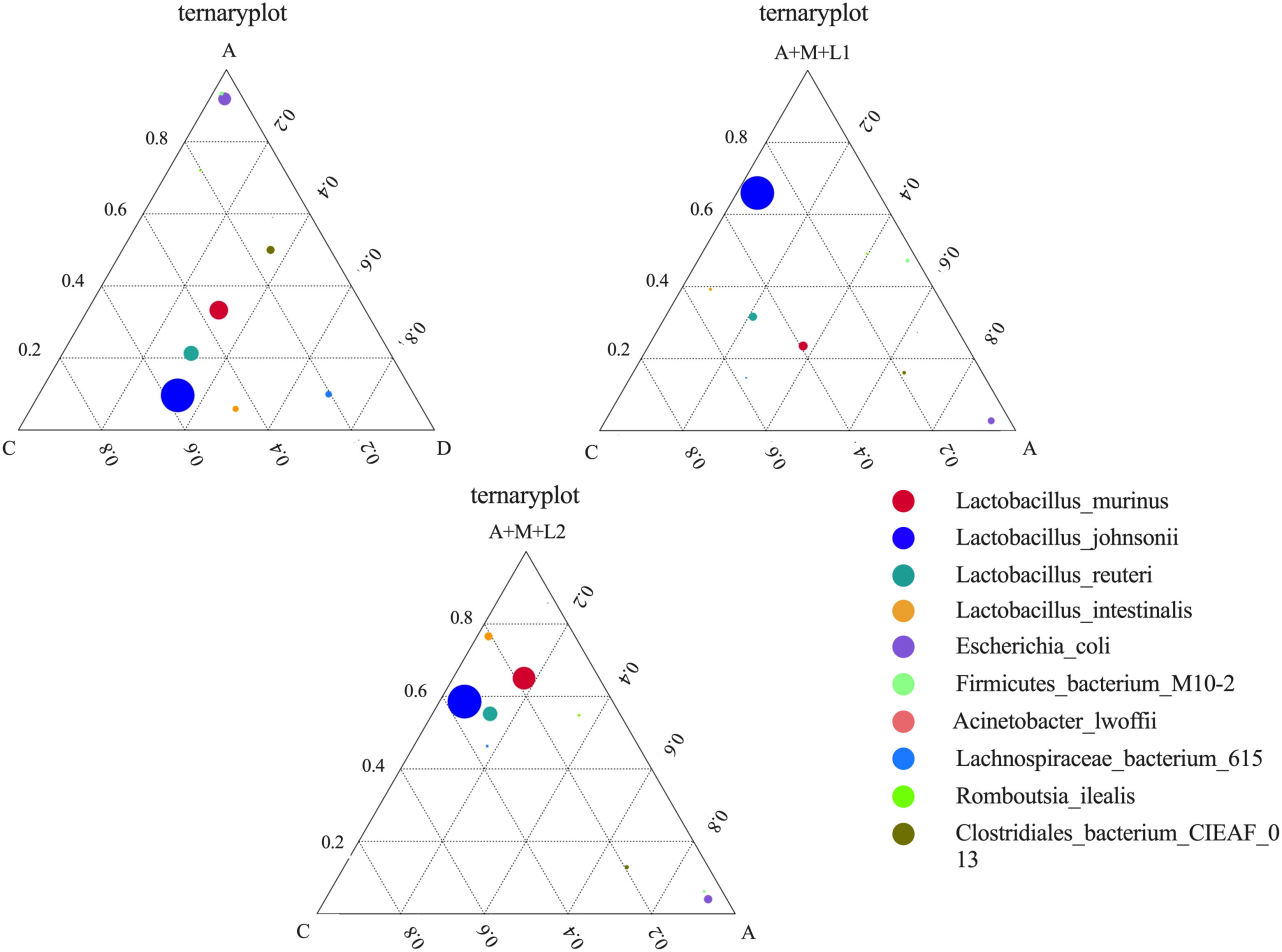
Differences in dominant species at the species level in each group's cecal flora.

To examine the variations in dominant species among the three sample groups at the species level, we identified the top 10 species sorted by average abundance for each of the three groups and created a ternary plot (Figure 10). The predominant bacterial populations in Groups C and D are dominated by LAC and *Lactobacillus johnsonii*, whereas Group A is predominantly comprised of *Escherichia coli*. Following the treatment of the mice, *Lactobacillus johnsonii* emerged as the predominant bacterial community in Groups A+M+L1 and A+M+L2, and the quantity of *Escherichia coli* diminished. Notably, Group A+M+L2 demonstrated a more significant effect.

### 3.10 Concentration of AFB_1_ in the feces

**Figure 11:**
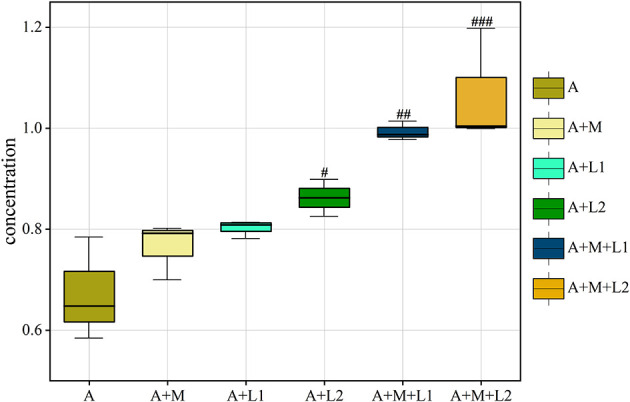
AFB_1_ in mice feces is shown as box plots with IQR (25th−75th percentiles), 1.5 × IQR whiskers, and median line. Symbols ^#^, ^##^, ^###^*P* < 0.05, 0.01, 0.001.

We also analyzed the AFB_1_ content in mouse feces across treatment groups (Figure 11). Fecal AFB_1_ was unchanged in A+M and A+L1 (*P* > 0.05), increased in A+L2 (*P* < 0.05), and highly elevated in A+M+L1 and A+M+L2 (*P* < 0.01; *P* < 0.001 vs. A).

## 4 Discussion

AFB_1_, as a highly toxic compound, is the most toxic of the known AFs. Oral intake of AFB_1_-contaminated food or feed, along with direct contact via skin or mucous membranes, represents the primary exposure pathways to AFB_1_ (32). As documented in the literature, both exposure routes exhibit harmful effects on the gastrointestinal and hepatic systems, as supported by epidemiologic and animal experimental evidence (33, 34). Certain bacteria, such as LAB, including *Bifidobacterium* and *L.plantarum*, can degrade AFB_1_ toxicity (35, 36). However, most studies focus on direct toxicity reduction by LAB while neglecting their complex host-colonization ability and unstable intestinal survival (37), which may limit their efficacy. Using MMT as a carrier, LAB-MMT complexes alleviate AFB_1_-induced immunotoxicity and oxidative stress while enhancing LAB's gastrointestinal survival (38, 39). However, there have been no reports on the combination of LGG/LAC-MMT to degrade AFB_1_. LGG/LAC-MMT protective effects against AFB_1_ toxicity in mice inform poisoning management.

AFB_1_ can be activated in the liver and bind to DNA and proteins, thus disrupting liver morphology (40, 41). ALT and AST are validated liver injury markers (42). MDA is a recognized oxidative damage biomarker, while T-AOC, SOD, CAT, and GSH-Px indicate oxidative stress status (43, 44). AFB_1_ induced hepatic histological lesions in mice, increasing ALT, AST, IL-1β, TNF-α, and MDA, while decreasing TP, ALB, PA, and antioxidant enzyme activities (SOD, CAT, GSH-Px). These findings confirm AFB_1_-mediated liver injury, inflammation, and oxidative stress. Single LGG, LAC, or MMT treatments only reduced MDA. Notably, the LGG/LAC-MMT combination mitigated liver injury, lowering MDA, transaminases (ALT, AST), and proinflammatory cytokines, while enhancing SOD, GSH, and CAT levels. This indicates that the combination of LGG/LAC-MMT can reduce the hepatotoxicity of AFB_1_ by significantly reversing liver injury, alleviating hepatic dysfunction and oxidative stress injury, as well as regulating the production of cytokines. Among them, the effect of LAC-MMT was more significant.

AFs cause intestinal lesions in animals, disrupt intestinal barrier function and modulate immune responses (45, 46). A related study in rats showed that AFB_1_ exposure induced duodenal mucosal/submucosal hemorrhage and oxidative stress (47). The present study agreed with other studies that AFB_1_ exposure caused structural abnormalities in mouse jejunal tissues, with significantly elevated levels of MDA, SOD, and GSH, suggesting that mice's jejunal tissues were damaged and accompanied by oxidative stress. LGG, LAC and MMT alone reduced the degree of tissue damage in AFB_1_-intoxicated mice, suggesting that all three have a protective effect on intestinal tissue damage in AFB_1_-intoxicated mice. In the past few decades, the oxidative stress coping mechanisms of LABs, their antioxidant potential, and their health-benefiting effects have been intensively examined. For example, *Lactobacillus fermentum* strain JX306 was found to significantly reduce MDA levels and increase GSH-Px, Glutathione S-transferase (GST) and SOD levels in the small intestine (48). In this study, LGG, LAC and MMT could up-regulate the levels of SOD, CAT, GSH and reduce the MDA content in the intestinal tissues of AFB_1_-intoxicated mice either by individual intervention or by combined application, in which the effect of combination of LGG/LAC-MMT was more significant. Thus, the LGG/LAC-MMT combination provided stronger protection against AFB_1_-induced oxidative damage, with LAC-MMT exhibiting the most pronounced effect.

Gut microbes are essential for livestock health and efficiency. AFB_1_ exposure damages host organs and depletes intestinal microbiota (49). 16S sequencing shows that AFB_1_ alters mouse gut microbiome composition. Flora diversity analysis showed significant decreases in ACE and Chao1 indices in the AFB_1_ group, indicating altered intestinal flora abundance and diversity. The two largest phyla levels that make up the intestinal microbiome are the *Firmicutes* and *Bacteroidota*. The proportion of thick-walled to anabolic bacilli has been linked to various pathological states, including intestinal metabolic balance and inflammatory marker levels (50). At both phylum and order levels, AFB_1_ exposure significantly reduced *Lactobacillales* abundance. It was hypothesized that AFB_1_ can trigger related diseases, such as oxidative stress and inflammatory responses, by decreasing the dominant flora in the intestines of mice. Studies have shown that AFs increase penetration and translocation of pathogenic bacteria (51–53). AFB_1_ decreased dominant gut bacteria and increased pathogens (e.g., *Escherichia coli*) in mice, consistent with prior findings. It suggests that AFB_1_ can lead to intestinal flora disruption, which can result in intestinal damage. The study confirmed that LABs have the function of regulating the balance of intestinal microbiota. LABs have been shown to alleviate intestinal microbiota dysbiosis, alter dominant intestinal species, and improve intestinal barrier function (54, 55). Additionally, the LAC-MMT combination exhibited superior effects on intestinal flora and barrier function compared to single components (56). In this study, ACE, Shannon, Simpson, and Chao1 indices were significantly higher in the LAC/LGG-MMT combination group than in other groups. This suggests that the LAC/LGG-MMT combination exhibits more pronounced effects on the richness and diversity of mouse intestinal flora. The LGG/LAC-MMT combination increased dominant flora and reduced pathogenic bacteria in mouse intestines. Among them, LAC-MMT co-application increased the colonization ability of LAC. To investigate AFB_1_ degradation, fecal AFB_1_ content was measured, showing that LGG, LAC, and MMT bound AFB_1_ and reduced intestinal absorption, with LAC and LAC/LGG-MMT being most effective. This indicates their ability to prevent intestinal AFB_1_ absorption. Although we did not observe any toxic effects of MMT combination therapy on mice during the 4-week trial period, different types and doses of MMT may produce different effects. Therefore, the long-term safety of MMT combination therapy requires further investigation.

## 5 Conclusions

This study revealed that the LGG/LAC-MMT combination potently alleviated AFB_1_-induced hepatic and intestinal tissue damage. Additionally, this combination alleviated both the inflammatory response and oxidative stress damage in these organs. Moreover, LGG/LAC-MMT played a crucial role in rebalancing the intestinal flora of AFB_1_-intoxicated mice. By enhancing the prevalence of dominant bacteria and reducing the population of pathogenic bacteria, it exerted a clinically significant protective function. Among these, the LAC-MMT combination emerged as the most effective treatment, which provides essential scientific evidence for the subsequent formulation development targeting AFB_1_ control.

## Data Availability

The original contributions presented in the study are included in the article/supplementary material, further inquiries can be directed to the corresponding authors.
